# Evaluation of the Prevalence of Complete Isthmii in Permanent Teeth Using Cone-Beam Computed Tomography 

**DOI:** 10.22037/iej.v12i4.17175

**Published:** 2017

**Authors:** Sina Haghanifar, Ehsan Moudi, Zahrasadat Madani, Foroozan Farahbod, Ali Bijani

**Affiliations:** a *Oral Health Research Center, Institute of Health, Department of Oral and Maxillofacial Radiology, Dental School, Babol University of Medical Sciences, Babol, Iran; *; b * Dental Materials Research Center, Institute of Health, Babol University of Medical Sciences, Babol, Iran; *; c * Student Research Committee, Babol University of Medical Sciences, Babol, Iran; *; d * Social Determinants of Health Research Center, Health Research Institute, Babol University of Medical Sciences, Babol, Iran*

**Keywords:** Cone-Beam Computed Tomography, Root Canal Anatomy, Root Canal Isthmus

## Abstract

**Introduction:**

The current study aimed at determining the prevalence of complete isthmii in permanent teeth, using cone-beam computed tomography (CBCT) in a selected Iranian community.

**Methods and Materials:**

In this cross sectional study, 100 CBCT images (from 58 female and 42 male patients) including 1654 teeth (809 maxillary and 845 mandibular teeth) were evaluated. Each tooth root was evaluated in axial plane (interval, 0.1 mm; thickness, 0.1 mm) from the orifice to the apex and from the apex to the orifice to detect the presence of complete isthmus. Scans of teeth with complete isthmii were reevaluated in axial, sagittal, and coronal planes with the thickness, 0.1 mm. Presence and absence of complete isthmii in each tooth was reported. The root canal was divided into 3 equal parts (cervical, middle and apical thirds), and isthmii were classified with respect to the start and end points. Findings were classified into 6 categories with respect to the start and end points of the isthmii: 1) the beginning and end in the cervical third; 2) the beginning in the cervical third and end in the middle third ; 3) the beginning in the cervical third and end in the apical third ; 4) the beginning and end in the middle third ; 5) the beginning in the middle third and end in the apical third and 5) the beginning and end in the apical third.

**Results:**

The prevalence of complete isthmus in permanent teeth was 8.6%, and the highest prevalence was reported in mesial roots of the mandibular first molars. In maxilla, the highest prevalence of complete isthmus was found in mesiobuccal roots of the maxillary first molars, whereas in canines and central incisors, no isthmii were detected. In the mandible, the lowest prevalence of isthmus was found in second premolars. In maxillary molars, isthmii starting and ending in the middle third of the root had the highest prevalence. On the other hand, isthmii in mandibular molars, from apical or middle third of the root beginning to the end of the apical third, had the highest prevalence.

**Conclusion:**

As the prevalence of complete isthmii was the highest in molars, endodontists should pay particular attention to accomplish a successful surgical or nonsurgical root canal therapy.

## Introduction

Irregularities in the root canal system, including isthmus, may limit the efficiency of endodontic instrumentations, irrigant solutions and intracanal medications [1-3]. An isthmus is a pulp space containing narrow extension, lateral interconnection or a transverse anastomosis in one or two root canals [4, 5]. Complete isthmus is a continuous connection between the two main canals of the same root, while partial isthmus is an incomplete connection with one or more openings between two main canals [6]. Isthmus classification described by Kim *et al.* [7] is as follows: incomplete isthmus which is a faint communication between two canals (Type I), definite connection between two canals which are considered complete isthmus (Type II), very short complete isthmus between two canals (Type III), complete or incomplete isthmus between 3 or more canals (Type IV) and two or three canal openings without visible connection.

Isthmus in the root canal system may include necrotic debris, tissue remnants, or organic layers, which contribute to the growth of microorganisms and lead to endodontic treatment failure[6, 7] . Therefore, knowledge of the root canal anatomy is a prerequisite for complete cleaning of the root canal and successful endodontic treatment [4, 8]. So far, several methods are applied to examine the morphology of the root canal including periapical radiography, vertical and cross sectional slicing, stereomicroscopy, surgical microscopy, dissecting microscopy, plastic casts, staining, clearing, electron microscopy, cone-beam computed tomography (CBCT) and micro-CT [4, 6, 7, 9-20]. However, considering the buccolingual orientation of small isthmii, they cannot be detected on radiographs before endodontic treatments [5].

The most common method is visual evaluation of sectioned teeth by microscopy, although optical magnification might not be sufficient, even in surgical or stereomicroscopes [4, 21]. In the study of Tabrizizadeh *et al.* [22] and Mehrvarzfar *et al.* [23] they evaluated the incidence and position of root canal isthmii in mandibular first molars using stereomicroscope. However, in recent years, new techniques, such as cone-beam computed tomography (CBCT), are used to evaluate the root canal anatomy [11-20]. Through CBCT, different types of teeth can be evaluated in axial, sagittal and coronal planes* in vivo*, and the morphologies of the root canals, *e.g.* isthmus which are not visible on two dimensional radiographs due to superimposition, can be detected. If available, CBCT can be used in endodontic treatment to visualize the root canal. Since management of complete isthmii is easier than that of partial ones [24], knowledge of their prevalence can play an important role in the success of root canal treatments. The prevalence of isthmus, similar to other anatomical irregularities of the root canal, depends on race of the population under study. With this background in mind, the current study aimed to determine the prevalence of complete isthmus in permanent teeth, using CBCT in a selected Iranian community.

## Materials and Methods

In the current cross sectional study, 100 CBCT images of 58 female and 42 male patients who were referred to a dental radiology service of a private clinic in Sari, Iran from January 2015 to January 2017 including 1654 teeth (809 maxillary and 845 mandibular teeth) were evaluated. The inclusion criteria was teeth with a fully developed root. On the other hand, the exclusion criteria were as follows: 1) third molar teeth, 2) root canal-treated teeth, 3) post-retained crowns, 4) external root resorption, 5) internal root resorption, 6) history of orthodontic treatment, 7) developmental problems and 8) pathological lesions. 

All images were acquired using Cranex™ 3D unit (Soredex, Helsinki, Finland) with the following settings: thickness, 0.1 mm; 6×8 cm field of view; 0.2 mm voxel size; 89 kVp tube voltage; 6 mA tube current all in high-resolution mode. Images were examined with the scanner’s proprietary software Ondemand 3D Dental Viewer software (Cybermed Inc, Irvine, CA) and TFT LED Monitor with full HD resolution 1920×1080 pixels (Lenovo Corp., NC, USA). 

In axial images, any continuous opening between two main root canals was considered as a complete isthmus. In each patient, the anterior teeth and premolars were evaluated, followed by the first and second molars. In maxillary first molars, the morphology of mesiobuccal root was assessed, followed by distobuccal and palatal roots. In addition, the mesial and distal roots were examined in mandibular molars, respectively. 

**Figure 1:**
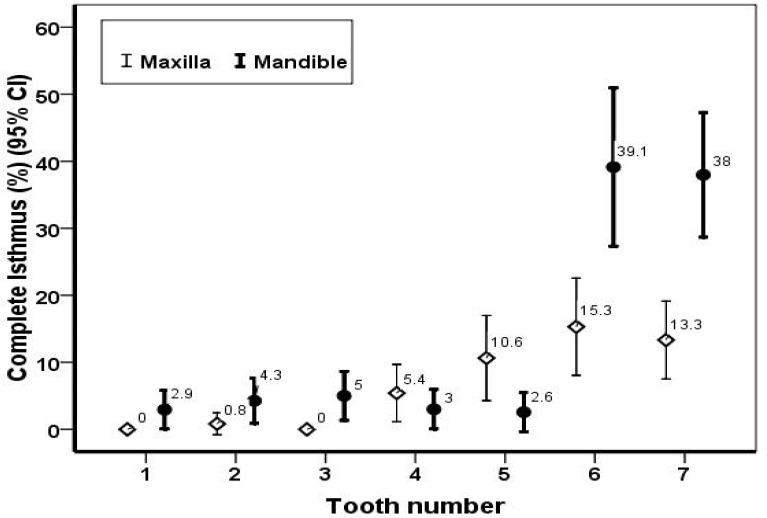
The prevalence of complete isthmii in permanent teeth

Each root was evaluated in axial plane (interval, 0.1 mm; thickness, 0.1 mm) from the orifice to the apex and from the apex to the orifice to detect the presence of complete isthmus (Figure 1). Scans of teeth with complete isthmii were reevaluated in axial, sagittal and coronal planes with 0.1 mm thickness. Presence and absence of complete isthmus in each tooth was reported. Then, the length of each root was divided into 3 equal sections (cervical, middle and apical thirds) and the findings were classified in 6 categories with respect to the start and end points of the isthmii [11, 19].

The beginning and end in the cervical third (CT) (CT-CT); The beginning in the cervical third and end in the middle third (MT) (CT-MT); The beginning in the cervical third and end in the apical third (AP) (CT-AT); The beginning and end in the middle third (MT-MT); The beginning in the middle third and end in the apical third (MT-AT); and The beginning and end in the apical third (AT-AT).

All images were evaluated by 2 experienced maxillofacial radiologists and the results were reported based on a consensus among radiologists.

Frequency and percentage were used to present the categorical values for this study and quantitative values were presented as mean±SD. Frequencies with confidence intervals (95% CI) were given. Analysis using the *Chi* square test, and the Yates correction or the Fisher exact test was done for the quantitative values. Student *T* test was used to compare these variables for independent samples. The significant level was set at 0.05. SPSS software version 17.0 (SPSS, Chicago, IL, USA) was used for this purpose.

## Results

The overall prevalence of complete isthmus in permanent teeth was 8.6%, with the highest prevalence attributed to mandibular molars. In the maxilla, the highest prevalence of complete isthmus was found in the first molars, followed by the second molars (mesiobuccal roots accounted for 93% of isthmii). The lowest prevalence was observed in the central teeth and canines (no isthmii). In the mandible, the highest prevalence was reported in the first molars, followed by the second molars, with the highest frequency reported in the mesial roots (92% and 90% in the mesial root of the first and second molars, respectively) and the lowest frequency in the second premolars. 

**Figure 2:**
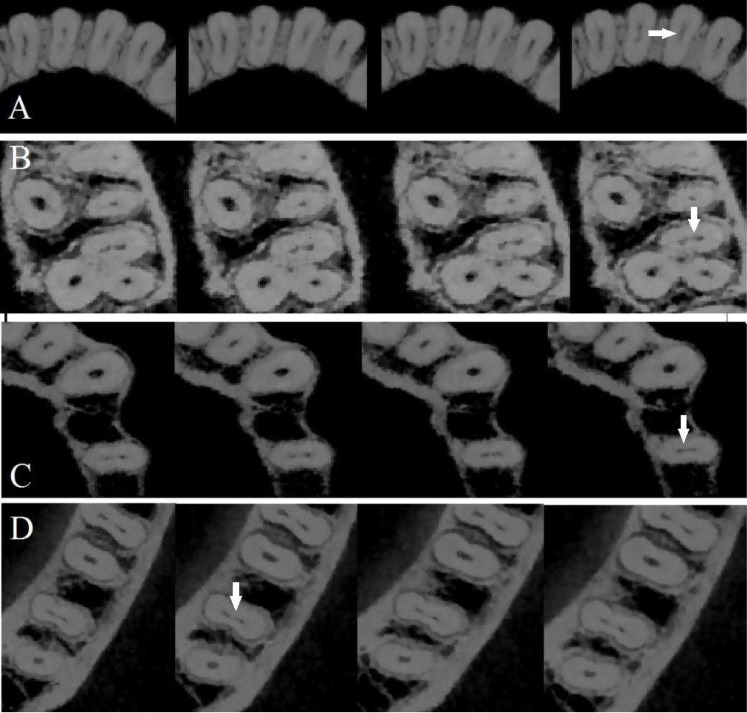
*A) *Complete isthmuses in mandibular central incisor; *B)* maxillary second molar; *C)* maxillary second premolar; *D)* mandibular second molar viewed in CBCT axial slices (interval, 0.1 mm; thickness, 0.1 mm) from coronal to apical direction. Arrows indicate isthmus

The prevalence of complete isthmus in the upper and lower teeth with respect to gender is presented in Table 1. There was no significant difference among males and females in terms of the prevalence of isthmii, except for the mandibular canines. Based on the findings, the prevalence of isthmii decreased with advancing age; however, the difference was not statistically significant. Mean age of patients with isthmii was 33.1±11.6 and for patients without isthmii was 34.6±10.8 (*P*=0.116). The prevalence of complete isthmus in permanent teeth is demonstrated in Figure 2 (95% CI).

Moreover, with respect to the beginning and end points of isthmus, the majority of reported cases in the maxillary molars began and ended in the middle third of the root (60% in maxillary first molars and 39% in maxillary second molars). The high frequency of isthmii was observed in mandibular molars, which began at the apical or middle third of the root and ended in the apical end. The prevalence of complete isthmus based on the beginning and end points is shown in Table 2.

## Discussion

In the current study, the highest prevalence of complete isthmii in permanent teeth was reported in the mesial root of mandibular first molars (36%), followed by the mesial root of second molars (34%), and mesiobuccal root of maxillary first molars (14.3%); these findings could be attributed to the high prevalence of two canals in these roots. Previous studies also reported high prevalence of isthmus in the mesial root of mandibular first molars and mesiobuccal root of maxillary first molars; however, the prevalence varied in different studies [4, 6, 11, 14-16, 19].

**Table 1:** The prevalence N (%) of complete isthmus in the upper and lower teeth in terms of gender

		**Central**	**Lateral**	**Canine**	**First premolar**	**Second premolar **	**First molar **	**Second molar**
**Maxilla**	**Female**	0	1 (0.8)	0	4 (3.6)	4 (4.20	8 (8.2)	8 (5.9)
	**Male**	0	0	0	2 (1.8)	6 (6.4)	7 (7.1)	10 (7.4)
	**Total**	0	1 (0.8)	0	6 (5.4)	10 (10.6)	15 (15.3)	18 (13.3)
	***P*** ** value**	-	1.000	-	1.000	0.161	0.239	0.068
**Mandible**	**Female**	2 (1.45)	5 (3.6)	7 (5)	3 (2.25)	1 (0.9)	17 (24.6)	25 (23.2)
	**Male**	2 (1.45)	1 (0.7)	0	1 (0.75)	2 (1.7)	10 (14.5)	16 (14.8)
	**Total**	4 (2.9)	6 (4.3)	7 (5)	4 (3)	3 (2.6)	27 (39.1)	41 (38)
	***P*** ** value**	0.625	0.413	0.045	1.000	0.292	0.283	1.000

**Table 2:** The prevalence of complete isthmus in permanent teeth with respect to the begining and end points

	**CT-CT**	**CT-MT**	**CT-AT**	**MT-MT**	**MT** **-** **AT**	**AT-AT**	**Total**	**No Isthmus**
**U1**	0	0	0	0	0	0	0 (0)	124 (100)
**U2**	0	0	0	1	0	0	1 (0.8)	120 (99.2)
**U3**	0	0	0	0	0	0	0 (0)	126 (100 )
**U4**	2	0	0	4	0	0	6 (5.4)	105 (94.6 )
**U5**	1	2	2	4	1	0	10 (10.6)	84 (89.4 )
**U6**	1	4	0	9	0	1	15 (15.3)	83 (84.7)
**U7**	1	5	0	7	4	1	18 (13.3)	117 (86.7)
**L1**	0	0	0	2	2	0	4 (2.9)	132 (97.1)
**L2**	0	0	0	4	0	2	6 (4.3)	135 (95.7)
**L3**	0	0	0	5	0	2	7 (5)	133 (95)
**L4**	0	1	0	3	0	0	4 (3)	129 (97)
**L5**	0	1	0	2	0	0	3 (2.6)	114 (97.4)
**L6**	1	4	2	4	8	8	27 (39.1)	42 (60.9)
**L7**	1	8	2	10	10	10	41 (38)	67 (62)

Similar to other anatomical features, including the number and morphology of roots, the number of canals, apical foramina, and ramification, the prevalence and site of isthmus vary among different people and depend on factors such as race, geographical region, age, gender, research methodology, sample size and the type of isthmus (complete or partial).

Estrela *et al [*19*].*, used CBCT and reported the highest prevalence of isthmus in the mesial root of mandibular molars, followed by the mesiobuccal root of maxillary first molars. The reported incidence rate in the current study was lower than their study, which could be due to the examination of only continuous connection between two canals in the same root; in other words, only complete isthmii were studied.

In the current study, the prevalence of complete isthmus in mandibular first molars was higher than the rate reported by Teixeira *et al. *[4] (22%). In microscopic studies, such as the one performed by Teixeira *et al. *[4], the prevalence of isthmii is examined *via* cross sectional slicing at specific distances from the apex, while the current study investigated the entire canal from the orifice to the apex; this is in fact one of the benefits of CBCT in studies on canal morphology.

In this study, the lowest prevalence of complete isthmus in permanent teeth was observed in maxillary anterior teeth; among the evaluated teeth, only one upper lateral tooth had complete isthmus. This could be due to the fact that the majority of maxillary anterior teeth have only one canal, and consequently, few studies are performed on the prevalence of isthmus in such teeth; moreover, all performed studies reported similar findings in line with those of the current study [19].

So far, many studies evaluated the prevalence of isthmus, using different methods. Most of these studies include microscopic and *in vitro* assessments, in which sectioning cause damage to the teeth; moreover, they produce a smear layer, which can fade out narrow isthmii and therefore affect the results [15]. The current study assessed the prevalence of isthmus using CBCT, which does not need cutting the tooth structure. Contrary to micro-CT, CBCT has a high resolution and can be performed in *in vivo* studies [25].

In the current study, the prevalence of complete isthmus was higher in maxillary second premolars than the first premolars; however, mandibular second premolars showed the lowest prevalence of complete isthmus among mandibular teeth. The prevalence of complete isthmus in the mandibular anterior teeth was 3% to 5%, which was lower than the prevalence reported by Estrela *et al. *[19], (mandibular central incisors, 33.3%; mandibular lateral incisors, 47.6%; and mandibular canines, 24%). Since Estrela *et al. *[19], reported both partial and complete isthmii, this difference can be justified, and it can be concluded that the prevalence of partial isthmus is considerable in such teeth.

In the current study, isthmii, which began from the middle third and ended in the apical third, and the ones which began and ended in the apical third, showed the highest prevalence in the mesial root of mandibular first and second molars; these findings were in line with those of a study by Pecora *et al. *[11]. In clinical situations, use of mechanical methods is very difficult to access and clean the above mentioned isthmii. 

In this study, complete isthmii, which began and ended in the middle third, had the highest prevalence in maxillary molar teeth; this finding was in contrast with the results reported by Pecora *et al*. [11] and Estrela *et al. *[19]. In addition to discrepant reports regarding prevalence of isthmii in permanent teeth, various sites of isthmus occurrence were also reported in previous investigations. Genetic and racial variations, as well as differences in the definition of isthmus, might have caused the discrepancies in the reported prevalence rates. The majority of performed studies were conducted in specific geographical regions *via* microscopic studies using cross sectional slices. In such studies, normally performed *in vitro*, there is no detailed information about the age or gender of the patients.

As mentioned earlier, CBCT is a useful method to evaluate the morphology of root canals *in vivo*. In *in vivo *studies conducted by CBCT, including the current research, age and gender were taken into considerations. Based on the findings of the current study, there was no significant relationship between the prevalence of isthmii and age or gender (except in mandibular canines); however, it should be noted that the subjects were not from diverse age groups.

In the current study, during the evaluation of root canal anatomy by CBCT, metal and streaking artifacts affected the image of the root canal and inhibited isthmus detection. However, smaller fields of view can reduce such artifacts and produce more accurate results. Analysis of partial isthmus prevalence by CBCT and comparing the results with those of the microscopic findings are recommended for future studies.

## Conclusion

As the prevalence of complete isthmii was the highest in molars, endodontists should pay particular attention to accomplish a successful surgical or nonsurgical root canal therapy.

## References

[1] Burleson A, Nusstein J, Reader A, Beck M (2007). The in vivo evaluation of hand/rotary/ultrasound instrumentation in necrotic, human mandibular molars. J Endod.

[2] Carr GB, Schwartz RS, Schaudinn C, Gorur A, Costerton JW (2009). Ultrastructural examination of failed molar retreatment with secondary apical periodontitis: an examination of endodontic biofilms in an endodontic retreatment failure. J Endod.

[3] Nair P, Henry S, Cano V, Vera J (2005). Microbial status of apical root canal system of human mandibular first molars with primary apical periodontitis after “one-visit” endodontic treatment. Oral Surg Oral Med Oral Pathol Oral Radiol Endod.

[4] Teixeira F, Sano C, Gomes B, Zaia A, Ferraz C, Souza‐Filho F (2003). A preliminary in vitro study of the incidence and position of the root canal isthmus in maxillary and mandibular first molars. Int Endod J.

[5] Mohammadzadeh Akhlaghi N, Khalilak Z, Vatanpour M, Mohammadi S, Pirmoradi S, Fazlyab M, Safavi K (2017). Root Canal Anatomy and Morphology of Mandibular First Molars in a Selected Iranian Population: An In Vitro Study. Iran Endod J.

[6] Weller RN, Niemczyk SP, Kim S (1995). Incidence and position of the canal isthmus Part 1 Mesiobuccal root of the maxillary first molar. J Endod.

[7] Kim S (2001). Color atlas of microsurgery in endodontics.

[8] Ingle JI (2008). Ingle's endodontics 6.

[9] Torabinejad M, Walton RE, Fouad A (2014). Endodontics-e-book: Principles and practice.

[10] Von Arx T (2005). Frequency and type of canal isthmuses in first molars detected by endoscopic inspection during periradicular surgery. Int Endod J.

[11] Pecora JD, Estrela C, Bueno MR, Porto OC, Alencar AHG, Sousa-Neto MD, Estrela CRdA (2013). Detection of root canal isthmuses in molars by map-reading dynamic using CBCT images. Braz Dent J.

[12] Li-na Z, Wen-hao Q, Jin H (2013). A cone-beam computed tomography study of changes in canal isthmus of maxillary first premolars before and after instrumentation. Shanghai Kou Qiang Yi Xue.

[13] Paqué F, Laib A, Gautschi H, Zehnder M (2009). Hard-tissue debris accumulation analysis by high-resolution computed tomography scans. J Endod.

[14] Fan B, Pan Y, Gao Y, Fang F, Wu Q, Gutmann JL (2010). Three-dimensional morphologic analysis of isthmuses in the mesial roots of mandibular molars. J Endod.

[15] Gu L, Wei X, Ling J, Huang X (2009). A microcomputed tomographic study of canal isthmuses in the mesial root of mandibular first molars in a Chinese population. J Endod.

[16] Mannocci F, Peru M, Sherriff M, Cook R, Pitt Ford T (2005). The isthmuses of the mesial root of mandibular molars: a micro‐computed tomographic study. Int Endod J.

[17] Matherne RP, Angelopoulos C, Kulild JC, Tira D (2008). Use of cone-beam computed tomography to identify root canal systems in vitro. J Endod.

[18] Endal U, Shen Y, Knut A, Gao Y, Haapasalo M (2011). A high-resolution computed tomographic study of changes in root canal isthmus area by instrumentation and root filling. J Endod.

[19] Estrela C, Rabelo LEG, de Souza JB, Alencar AHG, Estrela CR, Neto MDS, Pécora JD (2015). Frequency of root canal isthmi in human permanent teeth determined by cone-beam computed tomography. J Endod.

[20] Haghanifar S, Moudi E, Mesgarani A, Bijani A, Abbaszadeh N (2014). A comparative study of cone-beam computed tomography and digital periapical radiography in detecting mandibular molars root perforations. Imaging Sci Dent.

[21] Hsu Y-Y, Kim S (1997). The resected root surface The issue of canal isthmuses. Dent Clin North Am.

[22] Tabrizizadeh M, Sefat YK, Hakimian M (2014). In Vitro Stereomicroscopic Study of the Incidence and Position of Root Canal Isthmuses in Mandibular First Molars. Avicenna J Dent Res.

[23] Mehrvarzfar P, Akhlagi NM, Khodaei F, Shojaee G, Shirazi S (2014). Evaluation of isthmus prevalence, location, and types in mesial roots of mandibular molars in the Iranian Population. Dent Res J (Isfahan).

[24] Kim S, Kratchman S (2006). Modern endodontic surgery concepts and practice: a review. J Endod.

[25] Michetti J, Maret D, Mallet J-P, Diemer F (2010). Validation of cone beam computed tomography as a tool to explore root canal anatomy. J Endod.

